# F95. ASSESSING MANIC SYMPTOMS IN PATIENTS WITH SCHIZOPHRENIA USING THE YOUNG MANIA RATING SCALE

**DOI:** 10.1093/schbul/sby017.626

**Published:** 2018-04-01

**Authors:** Tae Yong Kim, Seon-Cheol Park, Joonho Choi, Han-Yong Jung, Joo Eon Park

**Affiliations:** 1 VHS Medical Center; 2 Inje University College of Medicine and Haeundae Paik Hospital; 3 College of Medicine, Hanyang University; 4 Soonchunhyang University Bucheon Hospital; 5 Keyo Hospital

## Abstract

**Background:**

Van Os and Kapur have proposed that the discrete categorical dichotomy of schizophrenia versus bipolar disorder be changed to a dimensional conceptualization. It is also known that manic symptoms can contribute to the clinical course and prognosis of schizophrenia. Hence, a domain for mania has been included in the Clinician-Rated Dimensions of Psychosis Symptom Severity (CRDPSS) in the Diagnostic and Statistical Manual of Mental Disorders, 5th edition (DSM-5). However, the psychometric properties of the Young Mania Rating Scale (YMRS) have been little studied in subjects with schizophrenia.

**Methods:**

One hundred and sixty-six inpatients with schizophrenia (diagnosed with DSM-5,2 age ≥ 18 years and ≤ 65 years, and length of hospital stay ≥ 2 weeks) were enrolled from two mental hospitals in Korea. The Institutional Review Board approved the study protocol, and informed consent was given by all study subjects before the start of the study. The Korean version of the YMRS was used to evaluate the severity of manic symptoms. In addition, the domain for mania in the CRDPSS was used to evaluate presence or absence of manic symptoms (0–1, absence; 2–4, presence).

**Results:**

The average age and age-at-onset of the subjects were 46.5 (SD = 11.2) and 25.2 (SD = 13.2) years, respectively. Half were men (51.5%), and most were unmarried (79.1%), religiously affiliated (61.5%) and educated below high school graduate level (73.0%). The mean chlorpromazine equivalent dose of prescribed antipsychotics was 921.1 (SD = 952.0) mg. The mean total score on the YMRS was 7.3 (SD = 6.9) and the mean item scores were: 0.2 (SD = 0.4) for elevated mood, 0.1 (SD = 0.4) for increased motor activity, 0.1 (SD = 0.4) for sexual interest, 0.1 (SD = 0.4) for sleep, 0.4 (SD = 0.8) for irritability, 0.6 (SD = 1.2) for speech, 0.8 (SD = 1.1) for language, 2.0 (SD = 3.3) for content, 0.2 (SD = 0.7) for aggressive behavior, 1.0 (SD = 1.0) for appearance, and 1.8 (SD = 1.7) for insight. The Cronbach α for the 11 YMRS items was 0.66, which is considered an acceptable level of internal consistency. Moreover, only 4% (n = 7) of the 166 subjects had manic symptoms as assessed by the mania domain in the CRDPSS. A receiver operating characteristic curve (ROC) showed that the optimal cut-off score distinguishing schizophrenia patients with and without manic symptoms was 10 with a sensitivity of 88.3% and specificity of 75.6% (area under curve = 0.803, P = 0.012).

**Discussion:**

Since a 10 point total score on the YMRS represents a mild level of Clinical Global Impression (CGI) severity of mania, we may conclude that our threshold on the YMRS for identifying manic symptoms in patients with schizophrenia is reasonable. Hence it may be useful to investigate the evaluation of manic symptoms in patients with schizophrenia from the perspective of deconstructing psychoses.

